# The Protective Effect against Extracellular Histones Afforded by Long-Pentraxin PTX3 as a Regulator of NETs

**DOI:** 10.3389/fimmu.2016.00344

**Published:** 2016-09-07

**Authors:** Kenji Daigo, Yuichiro Takamatsu, Takao Hamakubo

**Affiliations:** ^1^Department of Quantitative Biology and Medicine, Research Center for Advanced Science and Technology, The University of Tokyo, Tokyo, Japan; ^2^Humanitas Clinical and Research Center, Rozzano, Italy

**Keywords:** pentraxins, extracellular histones, cytotoxicity, coaggregation, sepsis

## Abstract

Pentraxin 3 (PTX3) is a soluble pattern recognition molecule that plays critical roles in innate immunity. Its fundamental functions include recognition of microbes, activation of complement cascades, and opsonization. The findings that PTX3 is one of the component proteins in neutrophil extracellular traps (NETs) and binds with other NET proteins imply the importance of PTX3 in the NET-mediated trapping and killing of bacteria. As NETs play certain critically important host-protective roles, aberrant NET production results in tissue damage. Extracellular histones, the main source of which is considered to be NETs, are mediators of septic death due to their cytotoxicity toward endothelial cells. PTX3 protects against extracellular histones-mediated cytotoxicity through coaggregation. In addition to the anti-bacterial roles performed in coordination with other NET proteins, PTX3 appears to mitigate the detrimental effect of over-activated NETs. A better understanding of the role of the PTX3 complexes in NETs would be expected to lead to new strategies for maintaining a healthy balance between the helpful bactericidal and undesirable detrimental activities of NETs.

## Introduction

The innate immune system serves as the first line of defense against pathogen invasion and consists of cellular and humoral arm. The innate immune response is triggered by pattern recognition molecules (PRMs) upon the recognition of pathogen-associated molecular patterns (PAMPs), which are structural patterns conserved across a broad spectrum of microbes (1, 2). In addition to PAMPs, PRMs recognize damage-associated molecular patterns (DAMPs) that are secreted from damaged cells as a “warning signal from the host” (2). Like the innate immune system, PRMs are classified into cellular and humoral components. The cellular arm of PRMs includes toll-like receptors (TLRs), C-type lectin receptors (CLRs), scavenger receptors, retinoic acid-inducible gene (RIG)-I-like receptors (RLRs) and NOD-like receptors (NLRs) with diverse patterns of localization, ligand recognition, and signal transduction. The humoral arm of PRMs includes complements, collectines, ficolins, and pentraxins that share a number of fundamental mechanisms against infection, such as complement activation, agglutination, neutralization, and opsonization (3, 4).

Pentraxin 3 (PTX3) was the first long pentraxin to be identified (5). The PTX3 gene is highly conserved across species (6). It is a circular multimeric glycoprotein that recognizes certain microbes and eliminates them through complement activation and opsonization. In addition to its activity in the innate immune system, PTX3 exerts effects in inflammation and matrix regulation (6). PTX3 expression includes hematopoietic and stromal cells by means of pro-inflammatory signals, while the characteristic expression mode of PTX3 production is that neutrophils store PTX3 in granules and release them in a “ready-to-release” manner (7). It is, thus, not surprising that PTX3 is one of the components of neutrophil extracellular traps (NETs) in which PTX3 serves as an anti-fungal activity. In addition to their host-protective activity, NETs also exert detrimental effects against the host. Extracellular histones, the major NET components and one of the DAMPs, afford a good example of the double-edged sword effect of NET components. They have bactericidal activity, but have also been shown to exert a cytotoxic effect on endothelial cells in sepsis. PTX3 has an ability to attenuate extracellular histone-mediated cytotoxicity through coaggregation (8). This implies that the role of PTX3 in NETs is not only to combat bacteria but also to mitigate the detrimental effects of NETs. Here, we describe our recent findings on the role of PTX3 as a regulator of NETs in relation to histone cytotoxicity.

## Pentraxin 3: General Description and Role in NETs

### Pentraxins

The pentraxins comprise an evolutionarily conserved multimeric protein family in which its members share the pentraxin domain [~200 amino acids (a.a.) long] in its C-terminal domain with a characteristic pentraxin signature (His–x–Cys–x–Ser/Thr–Trp–x–Ser, where x is any amino acid residue) (6, 9). The pentraxins are classified into two subfamilies based upon the N-terminal length, i.e., the short pentraxin and long pentraxin. C-reactive protein (CRP) and serum amyloid P component (SAP) are the prototypical short pentraxins, which are broadly known as acute phase proteins (10, 11). Neuronal pentraxin 1 (NPTX1) (12), neuronal pentraxin 2 (NPTX2) (13), neuronal pentraxin receptor (NPTXR) (14), and PTX4 (15) are the long pentraxins that have been reported in addition to PTX3.

### General Background of PTX3 (Genome, Expression, Structure, and Function)

#### Genome and Expression Pattern

The human PTX3 gene locates on chromosome 3q band 25, and consists of three exons and two introns. It consists of 1861 base pairs and is translated into 381 amino acids. The first and second exons encode the signal peptide (1–17 a.a.) and N-terminal domain (18–178 a.a.), and the last and third exon encodes the C-terminal pentraxin domain (179–381 a.a.), respectively. The promoter region of PTX3 contains PU-1, AP1, NF-κB, SP1, and NF-IL6, and the expression of PTX3 is triggered by certain primary inflammatory signals, such as TLR agonists, IL-1β, and TNFα.PTX3 is expressed several types of cells, including myeloid dendritic cells, peripheral blood leukocytes, macrophages, mononuclear phagocytes, vascular endothelial cells, smooth muscle cells, fibroblasts, adipocytes, glial cells, cumulus oophorus cells, mesangial cells, synovial cells, epithelial cells, and uroepithelial cells. Please refer to the cited reviews for the gene structure (3, 9) and expression pattern of PTX3 (3, 6) in more detail. In addition to the cells described above, lymphatic endothelial cells (16) and polymorphonuclear neutrophils (7) have distinct expression patterns. The former cells constitutively express PTX3, while the latter cells store the PTX3 protein in a “ready-to-release” manner, the details of which will be discussed below.

#### Protein Structure and Ligand Binding

After the processing of its signal sequence, PTX3 protein has an N-linked glycosylation of its Asn220 site (17, 18). The glycosidic moiety is important for the fine-tuning of the interaction with C1q and complement activation (17), the stabilization of Factor H binding (19), the interaction with M-ficolin (20), the interaction with P-selectin for attenuating neutrophil recruitment at sites of inflammation (21), and the blocking of the binding site of the influenza virus hemagglutinin (22).

**Figure 1:**
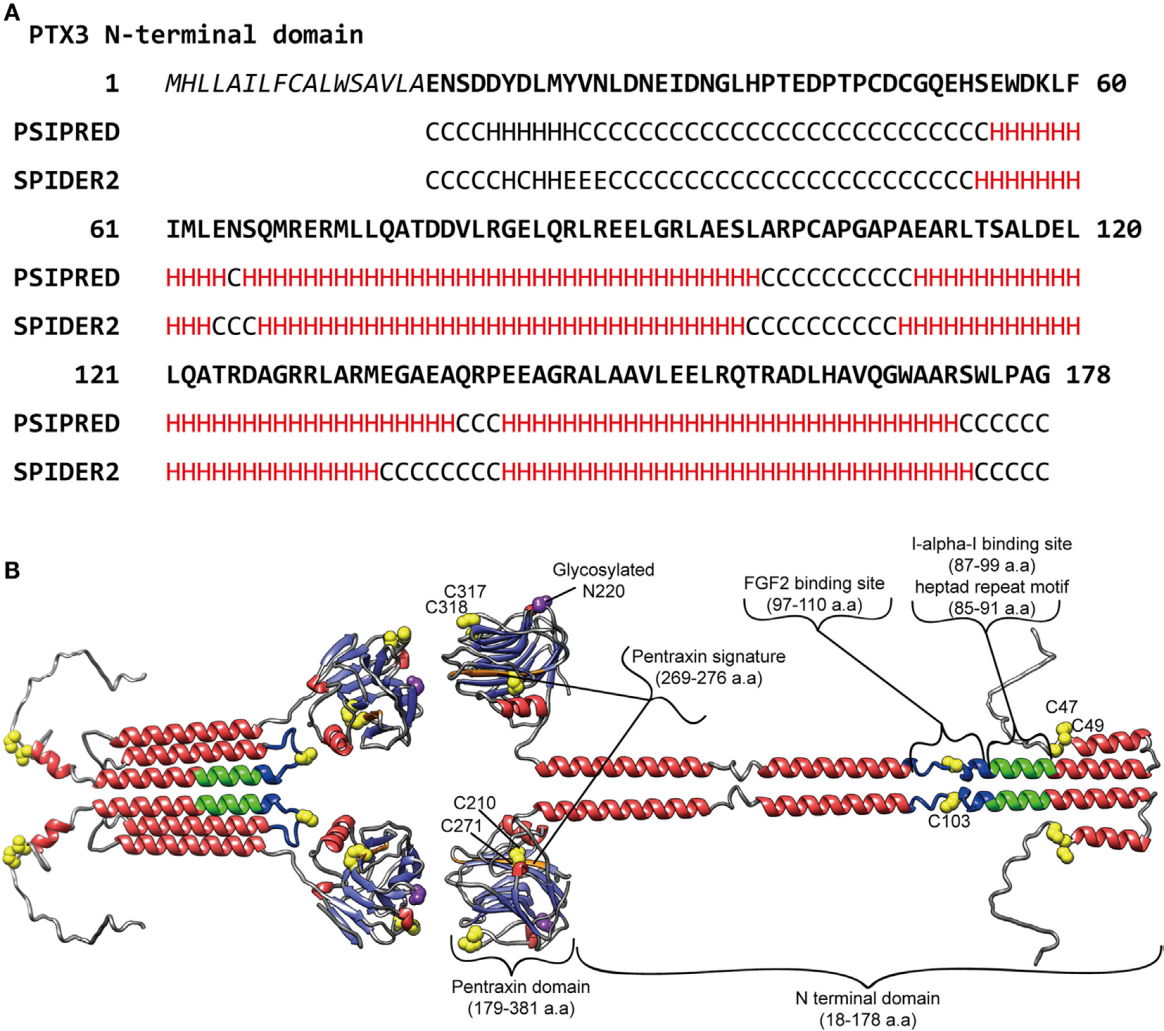
**Predicted molecular structure of PTX3**. **(A)** Amino acid sequence in the PTX3 N-terminus (1–178 a.a.) with the result of secondary structure predictions for each residue. H, alpha-helix; E, beta-sheet; C, random coil, respectively. The predictions were carried out using PSIPRED (30) and SPIDER2 (31). **(B)** A molecular modeling of PTX3 based on the result of the secondary structure predictions above as well as earlier studies (23–25, 28, 29). A half of the octamer consisting of asymmetric tetramer is shown. SWISS-MODEL (32) built of the pentraxin domain by homology modeling. Red, alpha-helix; light blue, beta-strand; yellow, cysteine residue contributes to multimer structure of PTX3; purple, glycosylated asparagine N220 in pentraxin domain; green, Inter-alpha-trypsin inhibitor (IαI) binding site, including heptad repeat motif; blue, FGF2 binding site, respectively. The drawing was obtained by USCF Chimera visualization software (33).

It is considered that, like the case of the short pentraxins, the PTX3 C-terminal pentraxin domain forms two antiparallel β sheets with a “jellyroll” topology (18, 23). As opposed to the PTX3 C-terminal domain, which is homologous among the pentraxins, the PTX3 N-terminal domain is unrelated to other known proteins. Presta et al. predicted four α-helix regions connected by short loops in the N-terminal domain using the PredictProtein server (24). They pointed out a heptad repeat motif (*abcdefg*) spanning a.a. residues 85–91, where *a* and *d* are non-polar residues and *e* and *g* are charged residues, together with hydrophobic residues repeated with a period of one every three to six a.a. in their helical regions (23) (Figure 1A). These motifs confer on PTX3 N-terminal domain a propensity for forming a coiled-coil structure. The signal of the α-helix was detected by circular dichroic (CD) spectroscopy (8). Inforzato et al. attributed the multimer formation of PTX3 to the inter- and intra-molecular disulfide bonds in the organization of the matrix (25) and proposed an asymmetric octamer structure for PTX3 based on biophysical analysis (26). PTX3 forms a circular-tetramer in the N-terminal domain, while the C-terminal domain forms octameric structure by stacking the two-tetramers. The N-terminal domain of one tetramer forms three intra-molecular helical coiled-coils, while the other, extended form of the tetramer consists of the inter-molecular interaction of the α-helixes (Figure 1B). These two types of tetramer consist of an octamer structure, thus rendering an asymmetry on PTX3. This multimerization is considered to be important for the interaction of a variety of PTX3 ligands (27) and the recognition of pathogens (28). The anti-PTX3 monoclonal antibody MNB4 that recognizes 87–99 a.a. of PTX3 inhibits PTX3-inter-alpha-trypsin inhibitor (IαI) interaction (29) and/or PTX3–FGF2 interaction. The minimal PTX3 N-terminal peptide required for interaction with FGF2 is 97–110 a.a. (24) (Figure 1B). Tetramer formation is required for FGF2 binding, and both types of tetramer can bind to FGF2 (26). We elucidated the tertiary structure of PTX3 by secondary structure prediction re-calculated with PSIPRED (30) and SPIDER2 (31) on the basis of the report by Presta et al. (23) (Figure 1A) with reference to the quaternary structure analysis reported by Inforzato et al. (26) (Figure 1B).

#### PTX3 in Innate Immunity, Inflammation, and Matrix Regulation

Pentraxin 3 exerts effects in the resistance against microbial, fungal, and bacterial infections through opsonization via the Fcγ receptor, complement regulation, and neutralization by direct recognition (3, 34, 35). PTX3 also regulates inflammation through complement [the classical complement component C1q (36), the alternative complement component Factor H (19), Factor H-related protein 5 (37) and C4BP (38), the lectin pathway ficolins (20, 39), and mannose-binding lectins (40)] and P-selectin interaction (21). Several studies have reported that PTX3 participates in the dynamic regulation of matrix formation. PTX3 knockout mice display female infertility due to cumulus matrix instability (41). The molecular mechanism underlying the phenotype is the interaction between PTX3 and the heavy chains (HCs) of IαI (29) as well as tumor necrosis factor α-induced protein 6 (TNFAIP6, also known as TSG6) (41). IαI and TSG6 are matrix component proteins that bind to hyaluronan. The direct interaction between PTX3 and these proteins builds up the super-molecular formation that contributes to proper cumulus matrix assembly. The PTX3 N-terminal binding site determined for IαI (29) is shown in Figure 1A. In tissue injury models, PTX3 knockout mice showed excessive fibrin deposition, clotting, and increasing collagen deposition (42). Further investigation revealed that fibrinogen/fibrin and plasminogen interaction with PTX3 promotes fibrinolysis (42). These findings on matrix component recognition imply the importance of PTX3 in the regulation of matrix formation as a hub molecule critical for appropriate higher-order structure. Interestingly, both the cumulus matrix formation and tissue remodeling defects induced by the lack of PTX3 were fond to be recapitulated by N-terminal domain PTX3 (41, 42), and the interaction of fibrinogen/fibrin and plasminogen with PTX3 was limited under an acidic condition (42). In contrast to the inhibitory activity of SAP in fibrosis, PTX3 promotes fibrocyte differentiation through FcγRI in fibrotic lesions (43). A detailed at the molecular level of these observations will result in a better understanding of matrix formation and the process of tissue remodeling supported by PTX3.

#### Expression and Role of PTX3 in Neutrophils

PTX3 expression is observed in neutrophil precursors but not matured neutrophils, while the PTX3 protein can be detected in both (7). No PTX3 expression is observed in eosinophils and basophils. PTX3 is stored mainly in specific granules, and partially in azurophilic and gelatinase granules (7, 44). In response to stimuli, such as microorganisms and TLR agonists, PTX3 is released into the extracellular space and localized in NETs. NETs comprise a mesh-like structure that consists mainly of DNA and histones (45). Some of the proteins derived from neutrophils are localized in NETs and are active in the trapping and killing of bacteria (46, 47). The PTX3 in NETs co-localizes with microorganisms and other NET component proteins (7, 44, 48). As it is expected that the complex formation of PTX3 with other proteins in NETs exerts synergistic antimicrobial effects, further investigation will be needed to fully understand the activities of the NET component proteins.

## Cytotoxic Effect of Extracellular Histones on the Endothelium in Sepsis

### The Extracellular Histones: A Double-Edged Sword in NETs

Histones are highly basic proteins that bind to DNA in the nucleus. Nuclear DNA becomes tightly condensed with the help of histones and ultimately forms chromosomes. Histones consist of five classes; H2A, H2B, H3, and H4 are the core histones, while H1 and H5 are linker histones (49). As well as the crucial functions of histones in the intracellular space, the extracellular histones also play certain roles, especially of the toxic sort. The first extracellular role of histones to be reported was that they are toxic to microbes (50). The toxic activity of purified calf thymus histones against various types of microorganisms was reported, including *Escherichia coli* K-12, *Klebsiella pneumoniae* and strains of *Shigdlae, Salmonella typhimurium*, and *Pseudomonas*. The microbicidal effect of each member of the histones was reported by subsequently. Histone H4 was identified from human sebocyte extract as an antimicrobial protein candidate and was shown to exert antimicrobial activity against *Staphylococcus aureus* and *Propionibacterium acnes* (51). The histones H2A and H2B exert a lethal effect on *Leishmania amazonensis*, but histone H1 does not exert any leishmanicidal effect (52). However, in contrast to the report by Wang et al., histone H1 has been identified as a potential antimicrobial agent (53). Although as yet not fully described, these reports imply that histone members have different types of toxic activities against a variety of microorganisms.

In contrast to the host-defense role of the extracellular histones, only detrimental effects have been reported to date. Extracellular histones are also toxic to host cells (54). The cytotoxicity of extracellular histones toward a variety of cell types and organs has been reported (55). Similar to the toxic effect against microbes, different histone members-dependent toxicity to each cell type has been reported. In the case of histone-mediated cytotoxicity to endothelial cells, histone H3 and H4 are the major components involved (56). As opposed to endothelial cells, histone H1 exerts cytotoxic effect against leukemia cells by causing severe plasma membrane damage while it does not affect normal peripheral blood mononuclear cells and bone marrow cells (57), suggesting that histone H1 exerts its cytotoxicity through leukemia cell-specific membrane components. In line with the report by Class et al., only histone H1, not core histones, is toxic to cortical neurons (58). In accordance with the reports above, the contribution of extracellular histones to certain disease models [reviewed by Allam and colleagues (55)] is mostly related to tissue injury.

In addition to the toxic effect of extracellular histones, signaling pathway activation and platelet aggregation have been reported. In sterile inflammation and cellular injury models, extracellular histones are released, and these are protected by an anti-histone antibody or as observed in TLR2- and TLR4-knockout mice (59), suggesting that TLR2 and TLR4 act as extracellular histone receptors. Similarly, the extracellular histone–TLR9–MyD88 pathway has been also reported in a hepatic ischemia/reperfusion injury model (60), as shown by the inhibitory effect of an anti-histone antibody and as observed in TLR9- and MyD88-knockout mice. Additionally, histone-mediated NLRP3 inflammasome activation has been confirmed both *in vitro* and *in vivo* (61). Extracellular histones bind to platelets and induce platelet aggregation. Histones activate platelets, and the TLR2 and TLR4 pathways are involved (62). Histone induces platelet aggregation, some part of which is mediated through fibrinogen and αIIβ3-integlin. Interestingly, similar to the cytotoxic effect, histone H3 and H4 display higher levels of platelet activation and aggregation. Consistent with the reports above, an *in vivo* analysis revealed that histone infusion resulted in thrombocytopenia (63).

Considering the fact that histones are the most abundant NET components (46), the histones in NETs would be expected to exert a lethal effect on the microbes captured. In sepsis, NETs prevent the dissemination of microbes in order to be able to capture them (64). However, growing evidence suggests that NETs also inflict tissue damage. Indeed, NETs contribute to the pathogenesis of a number of diseases (65, 66), including sepsis (65, 67). Histone blockade has been shown to be effective in protecting against histone-delivered/histone-mediated cytotoxic effect (68) and in an acute lung injury model in which NETs contribute (69).

### Relevance of the Extracellular Histones to Sepsis

Sepsis is a life-threatening organ dysfunction caused by a dysregulated host response to infection (70–72). The innate immune response participates in the pathogenesis of sepsis. In the initiation of sepsis, PRMs recognize the PAMPs that are derived from invading microorganisms. Upon PAMP recognition, PRM signal transduction triggers the secretion of pro-inflammatory mediators from innate immune cells (73–75). On the progression of sepsis, the innate immune system becomes over-reactive. This leads to hypercytokinemia and the recruitment of neutrophils into infectious regions, eventually resulting in multi-organ failure (75). In addition to PAMPs, DAMPs contribute to the pathogenesis of sepsis (73). DAMPs also trigger PRM-mediated signaling in systemic inflammatory response syndrome (SIRS), including trauma, burns, ischemia, and hemorrhage (73, 74, 76). As one of the DAMPs, the extracellular histone-mediated cytotoxicity toward endothelial cells has emerged as one of the features of the pathogenesis of sepsis (55, 56). Extracellular histones are present in the plasma of patients with sepsis (77). Histone administration *in vivo* results in neutrophil margination, vacuolated endothelium, intra-alveolar hemorrhage, and macro- and micro-vascular thrombosis, all of which are similar to the events that take place in the pathogenesis of sepsis (56).

## Protective Role of PTX3 Against Extracellular Histones: Implications for the Maintenance of a Good Balance of NETs

**Figure 2:**
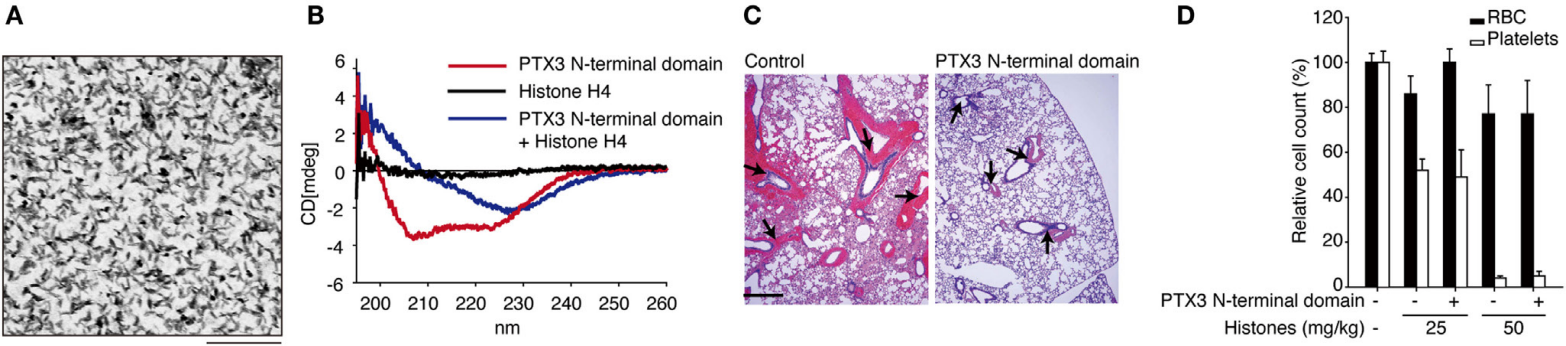
**Roles of PTX3 against extracellular histones**. **(A)** Electron microscopic image of PTX3–histones coaggregation. Scale bar: 0.2 μm. **(B)** CD spectra of PTX3–histone mixture. **(C)** *In vivo* protective effect of PTX3 administration against histone infusion-mediated hemorrhage in the murine lung. **(D)** Histone infusion-mediated thrombocytopenia was not rescued by PTX3 administration. All of the results were obtained from the paper by Daigo et al. (8).

**Figure 3:**
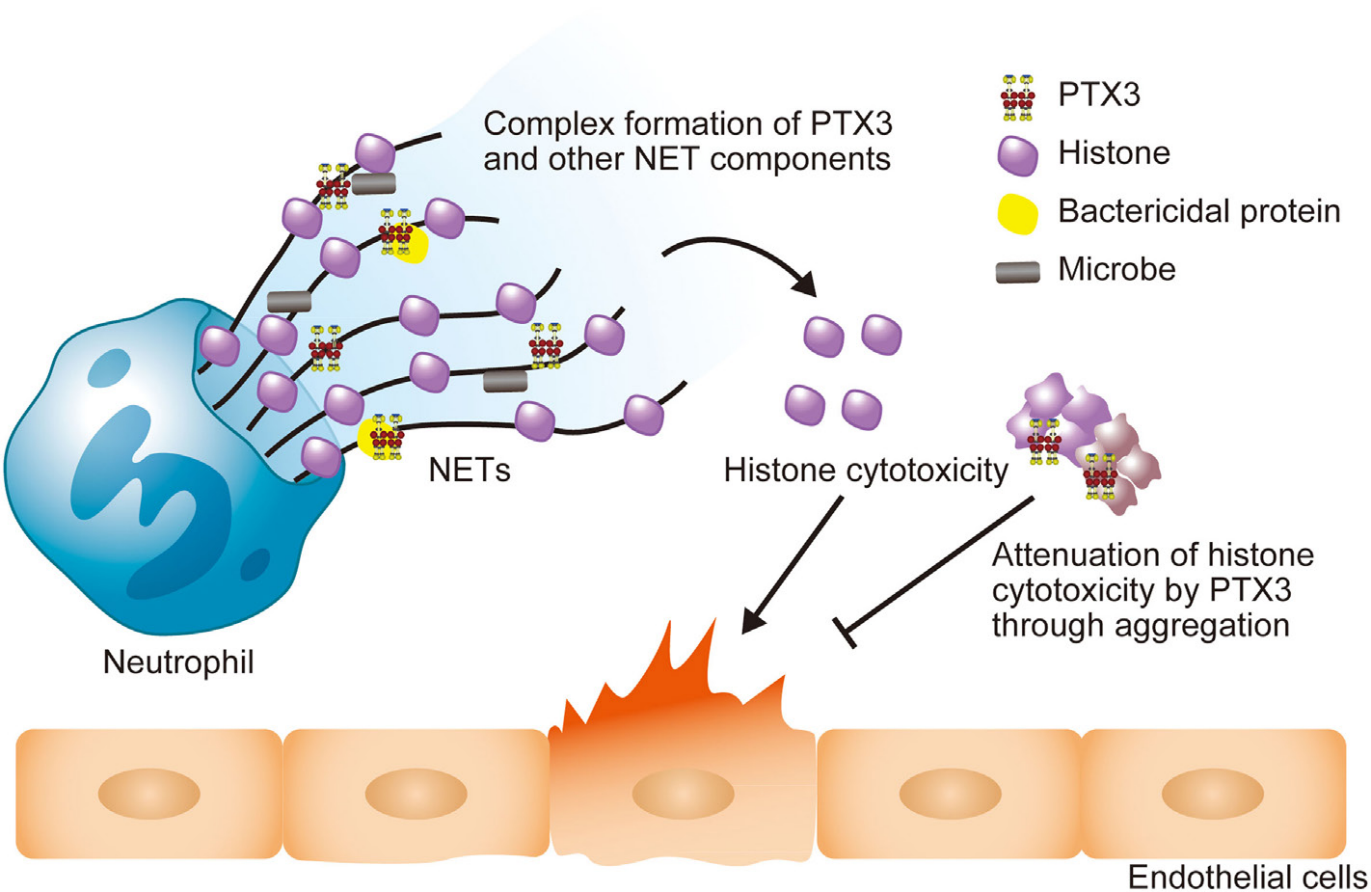
**Schematic illustration of the roles of PTX3 in NETs**. The PTX3 present in NETs forms a complex with other NET component proteins, including bactericidal proteins. This might enhance microbial clearance via synergistic effects. NET histones exert cytotoxicity toward endothelial cells, but PTX3 attenuates this cytotoxicity through aggregation. Thus, PTX3 function to maintain the balance of the beneficial and detrimental effects of neutrophils.

Extracellular histones are considered as a major factor in the severity of sepsis that results in organ failure and, thus, are targets in the treatment of sepsis. Certain inhibitors of the extracellular histone-mediated detrimental effects have been reported, such as activated protein C (APC), heparin, albumin, CRP, recombinant thrombomodullin (rTM), and IαI. The inhibitory effect against extracellular histones differs for each factor (Table 1). Extracellular histones were identified in a proteomic analysis of circulating PTX3 complexes in patients with sepsis (48). Considering the report that PTX3-transgenic mice are resistant to death from sepsis (78), the complex formation of PTX3 and histones is considered to have a host-protective in sepsis by attenuating extracellular histone-mediated detrimental effects. In the effort to understand the molecular mechanisms of PTX3–histone complex formation, both direct and high-affinity binding between PTX3 and histones has been reported. Of note, it was found that the binding induced coaggregation of PTX3 with histones due to a disorder of the PTX3 secondary structure (Figures 2A,B) (8). A cell-based assay revealed that PTX3 blocks histone-mediated cytotoxic activity toward endothelial cells. This blockade induced by PTX3 has been confirmed in all of the histone members. An *in vivo* analysis performed to investigate the function of PTX3–histone complex formation showed that PTX3 protects against histone-mediated endothelial cell cytotoxicity. The N-terminal domain of PTX3 was shown to be sufficient for both aggregate formation with histones and protection against histone cytotoxicity. This suggests the possibility of using the N-terminal PTX3 domain protein in sepsis treatment; indeed, *in vivo* administration resulted in resistance to septic lethality. Interestingly, however, the *in vivo* administration of PTX3 attenuated extracellular histones-mediated cytotoxicity, but did not suppress histone-mediated thrombocytopenia (Figures 2C,D). This result suggests that PTX3 has a distinct protective mechanism against histone-mediated detrimental effects because, among the factors reported, PTX3 is the only molecule that does not also protect against thrombocytopenia (Table 1). As it is considered that NETs are the source of extracellular histones, the protective activity of PTX3 against histone-mediated endothelial cell cytotoxicity implies that PTX3 participates in the regulation of NETs by attenuating the detrimental effects of NETs exerted by extracellular histones (Figure 3).

**Table 1:** **Inhibitors of extracellular histones**.

Inhibitors	Inhibitory effects on the pathogenic effects of extracellular histones					Reference
	Endothelium cytotoxicity	Platelet aggregation	Thrombocytopenia	Lung injury	Acute death	
APC	Inhibit	–	–	Inhibit	Inhibit	(56)
Heparin	Inhibit	Inhibit	Inhibit	Inhibit	Inhibit	(63, 79)
Albumin	Inhibit	Inhibit	–	–	–	(80, 81)
CRP	Inhibit	Inhibit	Inhibit	Inhibit	Inhibit	(82)
rTM	–	Inhibit	Inhibit	Inhibit	Inhibit	(83)
IαI	Inhibit	Inhibit	Inhibit	Inhibit	–	(84)
PTX3	Inhibit	–	No inhibitory effect	Inhibit	Inhibit	(8, 81)

*APC, activated protein C; CRP, c-reactive protein; rTM, recombinant thrombomodullin; IαI, inter-alpha-trypsin inhibitor; PTX3, pentraxin.3*.

## Perspectives and Concluding Remarks

Pentraxin 3 in NETs plays a variety of antimicrobial roles through pathogen recognition, complement regulation, and complex formation with other NET component proteins, including histones. Growing evidence supports the notion that PTX3 has a role in the regulation of extracellular histones, which are considered to be both diagnostic and therapeutic targets in certain severe diseases, including sepsis, due to their cytotoxicity and DAMP activity. PTX3 exerts a host-protective role against the histone-mediated detrimental effects that occur in sepsis. It is also expected that an elucidation of the detail of PTX3–histone aggregate formation will lead to new strategies for sepsis treatment. It is noteworthy that the matrix formation, tissue remodeling and aggregate formation induced by PTX3 are mainly associated with the N-terminal domain of PTX3. The N-terminal domain of PTX3 has the capacity to form a coiled-coil structure through heptad repeat motif with repeated hydrophobic residues. As a result of its activity of inter-molecular disulfide bond formation, PTX3 forms large complexes that interact with many different proteins. Such super-molecular formation with the assistance of PTX3 might be the ancestor of host-protective reactions. Further investigations are needed to elucidate the molecular mechanism of PTX3 complex interactions with proteins in NETs, especially histones.

## Author Contributions

KD, YT, and TH wrote the manuscript. YT performed secondary structure prediction and 3D structure modeling.

## Conflict of Interest Statement

The authors declare that the research was conducted in the absence of any commercial or financial relationships that could be construed as a potential conflict of interest.
